# Post-pandemic increase of Group A streptococcal infections in adults: a retrospective cohort study from 2012 to 2024

**DOI:** 10.1007/s15010-025-02668-9

**Published:** 2025-10-31

**Authors:** Anne Kathrin Lösslein, Tessa Goerne, Roland Elling, Tobias Wengenmayer, Georg Häcker, Paul Marc Biever

**Affiliations:** 1 Institute for Infection Prevention and Control, Faculty of Medicine, Medical Center - University of Freiburg, University of Freiburg, Freiburg, Germany; 2 Institute of Medical Microbiology and Hygiene, Faculty of Medicine, Medical Center - University of Freiburg, University of Freiburg, Freiburg, Germany; 3 Institute for Immunodeficiency, Center for Chronic Immunodeficiency, Faculty of Medicine, Medical Center - University of Freiburg, University of Freiburg, Freiburg, Germany; 4 Department of General Pediatrics, Adolescent Medicine and Neonatology, Faculty of Medicine, Medical Center-University of Freiburg, University of Freiburg, Freiburg, Germany; 5 Interdisciplinary Medical Intensive Care, Faculty of Medicine, Medical Center - University of Freiburg, University of Freiburg, Freiburg, Germany

**Keywords:** Group A streptococci, Adult patients, Intensive care unit, COVID-19 pandemic, Infection epidemiology, Post-pandemic infection trends, Public health measures

## Abstract

**Objectives:**

After the COVID-19 pandemic an increase in Group A streptococcal (GAS) infections was reported mostly for children in several countries worldwide. However, data on infections in adults remain limited. Therefore, we focus on the pre- and post-pandemic incidence of GAS infections in adults, the need for intensive care treatment and the microbiological sampling patterns.

**Methods:**

We performed a retrospective cohort study of hospitalized adult patients from 2012 to 2024, based on a positive GAS detection in their routine microbiological sampling. We compared the post-pandemic phase after lifting the isolation measures in April 2022 to the pandemic and pre-pandemic phase. Additionally, we analysed positive rapid assessment tests in children, indicative of the prevalence of GAS tonsillitis in this population.

**Results:**

In the post-pandemic months, we observed a significant increase in overall hospitalized adult patients (IRR 2.94) and ICU patients (IRR 2.5) with GAS infections. The rise can be attributed to an increase in both invasive and non-invasive GAS detections.

**Conclusions:**

The increase in GAS infections is not only relevant in paediatric patients, but also has significant relevance in adult patients. Physicians need to be aware of this increase. The data of 2024 show a sustained increase and an incidence that has not returned to pre-pandemic levels.

## Introduction

The COVID-19 pandemic due to SARS-CoV2 was accompanied by worldwide infection control measures, including wearing of masks and isolation measures. In Germany, several lockdowns starting from 2020 were an integral part of the public health measures. On April 3rd 2022, restrictions were lifted. The implemented measures had a long-term impact not only on the epidemiology of SARS-CoV2, but also on that of various other pathogens and diseases worldwide, some of which have become apparent only in retrospect. For instance, respiratory viruses showed atypical seasonality [1, 2], the tuberculosis incidence temporarily decreased during the pandemic [3] and *Mycoplasma pneumoniae* infections showed a delayed but significant re-appearance after the pandemic [4]. Group A streptococci (GAS) can cause a broad range of clinical symptoms, from pharyngitis to severe wound infections and toxic shock syndrome [5]. An increase in GAS infections has been reported after the pandemic, in particular in children [6, 7]. This increase is not limited to Scarlet fever but extends to invasive GAS (iGAS) infections [8]. Data on GAS infections in adult patients, in particular the incidence in the recent post-pandemic years, are limited [9]. We therefore conducted a retrospective analysis of pre- and post-pandemic GAS infections in adult patients receiving inpatient treatment at a tertiary-care hospital in southern Germany.

## Methods

### Study design and data collection

We conducted a retrospective cohort study of adult patients with detection of GAS in the routine microbiological diagnostics during their stay at the University Medical Center Freiburg from 2012 to 2024. The microbiological results with GAS detection per year including the time point of material collection and type of specimen were extracted from the laboratory software [M/lab] (DORNER Health IT Solutions). Patients who only received treatment in the outpatient clinics were excluded from the analysis. ICU/IMC (Intensive Care Unit/Intermediate Care Unit) treatments, length of hospital stay, outcome and epidemiological data (age and sex) were extracted from electronical health records. The microbiological specimens were categorized as invasive materials (blood culture, spinal fluid, tissue samples/biopsies, puncture material (aspirates), drain secrete, tracheal secretion, bronchoalveolar lavage, intraoperative swabs) and non-invasive materials (sputum, general swabs, urine). The local Data Integration Center (DIZ) provided the retrospective data of rapid antigen testing for GAS in children from suspected GAS tonsillitis cases, which were collected and analysed in house using Sofia^®^ Strep A + Fluorescent Immunoassay (FIA) (QuidelOrtho™) lateral-flow-assay with the Sofia^®^ 2 Analyzer (QuidelOrtho™) at the Center for Paediatrics at the University Medical Center Freiburg between April 2014 and November 2023. Data before April 2014 were not digitally available. Inconclusive test results were disregarded. In addition, we extracted the total number of microbiological test orders per year at the Institute of Medical Microbiology and Hygiene of the University Medical Center Freiburg.

### Statistical analysis

Statistical analyses were performed using RStudio (R version 4.3.3) and GraphPad Prism 10. Continuous variables were compared using unpaired two-tailed t-tests, and categorical variables using Fisher’s exact test. To account for differences in exposure time across periods, incidence rate ratios (IRRs) were calculated using Poisson regression. A piecewise linear regression analysis was performed to evaluate trend changes in positivity rates, referring to all requested microbiological analyses. A *p*-value < 0.05 was considered statistically significant; IRRs were reported with 95% confidence intervals.

## Results

**Fig. 1:**
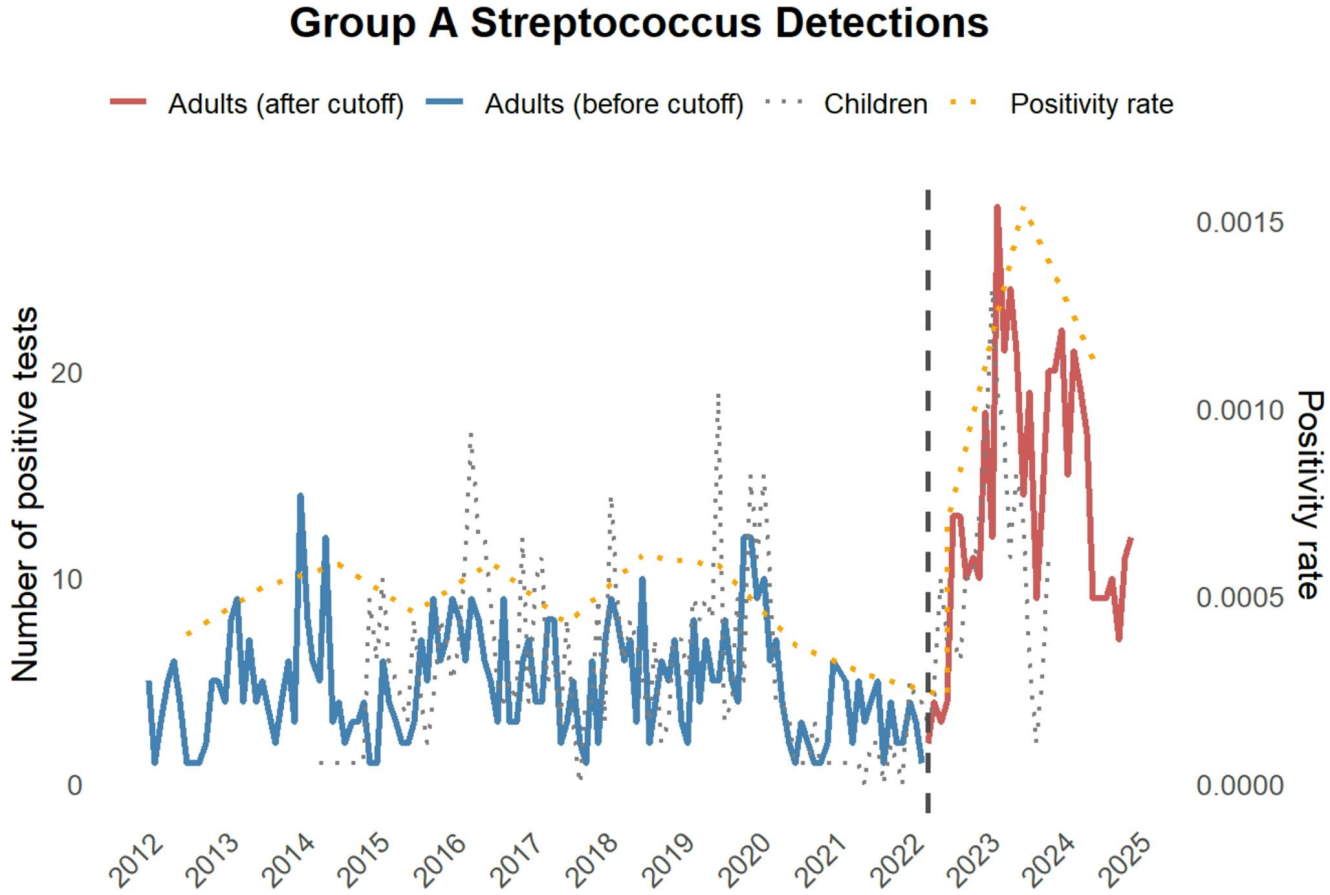
Group A *Streptococcus* detections in hospitalized adult patients in the routine microbiological sampling before (blue) and after (red) the relaxation of isolation and contact measures (3rd April 2022, dashed line). The grey dotted line represents rapid assessment testing in children in the emergency department and the yellow dotted line represents the GAS positivity rate compared to all microbiological tests requested

We included 1,034 case records of adult patients who received inpatient treatment at the University Medical Center Freiburg between 2012 and 2024, and in whom GAS had been detected by microbiological sampling in the course of normal care. There was a sharp increase in the incidence of GAS detection in 2023 and 2024 compared to previous years (Fig. 1). The GAS positivity rate remained stable from 2012 through early 2022 but showed a significant increase starting in April 2022. Piecewise regression revealed a statistically significant change in trend (*p* = 0.003), with a marked rise in positivity rates post cut-off. We compared our findings to the results of GAS rapid assessment tests (RAT) from oropharyngeal specimens performed in children. Here, a drop in 2021 became apparent. In this outpatient setting, an increase in positive RAT tests in children was seen in the post-pandemic phase (data available only till November 2023), which suggests uncomplicated GAS infections also increased. As most isolation measures in Germany were lifted on April 3rd, 2022 — with mask mandates remaining only in high-risk settings and public transportation — we defined this date as the beginning of the post-pandemic phase. We then compared the subsequent data to previous years, with a particular focus on cases requiring intensive care. Similar to a significantly increased incidence rate ratio of GAS infections in adults (2.94 fold, [2.56–3.33]), the need for ICU treatment among GAS patients increased (2.5 fold, [1.82–3.45]), (Table 1). Notably, there were no significant differences in median age, the rate of fatal outcomes or the median duration of the hospital stay (Table 1).

**Table 1:** Overview of epidemiological characteristics and the hospital stay of all adult patients and the subgroup of ICU patients separately before and after relaxation of isolation measures (cut-off date 3rd April 2022). The percentages refer to the respective patient numbers. * Test positivity rate calculated by piecewise linear regression analysis

	Total	Before cut-off date	After cut-off date	*P* value	Incidence Rate Ratio (CI)	*P* value
**Observation** **period (d)**	4748	3744	1003			
**all patient cases**	**1034**	**583**	**451**		2.94 (2.56–3.33)	**< 0.001**
all patient cases/1000 observations days	217.78	155.72	449.65			
Sex (f)	451 (43.6%)	253 (43.4%)	176 (39.2%)	0.162		
Age	45.89 (SD 17.46)	45.98 (SD 17.97)	45.78 (SD 16.8)	0.8519		
ICU	162 (15.67%)	97 (16.64%)	65 (14.41%)	0.3437	2.5 (1.82–3.45)	**< 0.001**
Outcome (death)	37 (3.6%)	20 (3.43%)	17 (3.77%)	> 0.999		
Length of hospital stay (d)	11.52 (SD 14.31)	11.7 (SD 12.43)	11.3 (SD 16.46)	0.6531		
patient cases with proof of GAS in invasive sample	581 (56.2%)	345 (59.18%)	236 (52.33%)	**0.0316**	2.56 (2.17–3.03)	**< 0.001**
**subgroup ICU**	**162**	**97**	**65**		2.50 (1.82–3.45)	**< 0.001**
ICU patient cases/1000 observations days	34.1	25.9	64.8			
Sex (f)	63 (38.9%)	34 (35.1%)	29 (44.6%)	0.2515		
Age	56.36 (SD 17.14)	56.78 (SD 16.89)	55.74 (SD 17.75)	0.7058		
Outcome (death)	34 (20.10)	18 (18.56%)	16 (24.6%)	0.4317		
Length of hospital stay (d)	23.57 (SD 22.76)	21.77 (SD 17.19)	26.26 (SD 28.96)	0.2212		
ICU patient cases with proof of GAS in invasive sample	133 (82.1%)	78 (80.41%)	55 (84.62%)	0.5372	2.63 (1.85–3.7)	**< 0.001**
**Total number of microbiological test requests**	1.617.043	1.225.379	391.664			
Detection of GAS per tests (positivity rate)	0.0006394	0.0004758	0.0011515	0.0032*		

We stratified the microbiological samples as invasive and non-invasive samples as illustrated in the method section. Both in the entire patient collective (2.56 fold, [2.17–3.03]) and the ICU subgroup (2.63 fold, [1.85–3.7]), we found a significantly increased incidence rate ratio for the detection of GAS in invasive samples (Table 1) after the pandemic. Nevertheless, the percentage of invasive samples was stable or even decreased (Table 2), which suggests that the rise in GAS infections also extends to GAS detections in non-invasive samples, typically associated with milder infections. The percentage distribution of detections in specific materials remained largely constant, with the exception of a reduction in intraoperative swabs in the overall cohort (Table 2).

**Table 2:** Details on the microbiological sampling with proof of GAS of the cohort in Table 1. The percentages refer to the patient numbers in Table 1

Microbiological sampling with proof of GAS	Total	Before cut-off date	After cut-off date	*P* value
**all patient cases** **(Table** 1)	**1034**	**583**	**451**	
Invasive sample numbers in all patient cases	699	401	298	0.1147
Blood culture	83 (8%)	45 (7.72%)	38 (8.43%)	0.7295
Tissue sample/ biopsy	152 (14.7%)	80 (13.72%)	72 (15.96%)	0.3306
Aspirate	93 (8.99%)	44 (7.55%)	49 (10.86%)	0.0791
Intraoperative swab	315 (30.46%)	199 (34.13%)	115 (25.50%)	**0.0027**
Deep respiratory specimen	47 (4.55%)	29 (4.97%)	18 (3.99%)	0.5475
Spinal fluid	2 (0.19%)	0	2 (0.44%)	0.1900
Drainage fluid	7 (0.68%)	3 (0.51%)	4 (0.89%)	0.4776
**subgroup ICU** **(**Table 1**)**	**162**	**97**	**65**	
Invasive sample numbers in ICU-subgroup	188	105	83	0.4209
Blood culture	41 (25.31%)	22 (22.68%)	19 (29.23%)	0.3623
Tissue sample/ biopsy	48 (29.63%)	30 (30.93%)	18 (27.69%)	0.7270
Aspirate	24 (14.81%)	13 (13.4%)	11 (16.92%)	0.6526
Intraoperative swab	39 (24.07%)	20 (20.62%)	19 (29.23%)	0.1950
Deep respiratory specimen	31 (19.14%)	18 (18.56%)	13 (20.0%)	0.8407
Spinal fluid	1 (0.62%)	0	1 (1.54%)	0.4012
Drainage fluid	4 (2.47%)	2 (2.06%)	2 (3.08%)	> 0.999

## Discussion

Our retrospective analysis highlights the increase of detected GAS infections after the COVID-19 pandemic not only in children but also in adult patients, similar to other reports, and with a particular focus on ICU admissions and the type of microbiological sampling. Interestingly, this increase in incidence was remained through 2024, with possibly a return to pre-pandemic patterns in 2025 (Fig. 1). We found an increase in GAS detections in both invasively and non-invasively collected microbiological samples. Although we detected an absolute increase in associated ICU admissions per 1,000 days after lifting the isolation measures, the relative proportion of ICU admissions was stable. This indicates that we do not only observe an increase of invasive and severe GAS infections in adults, but a general increase also of milder infections [10]. Higher virulence of the now circulating strains seems unlikely. The higher number of RAT tests in children in 2023 also suggests higher GAS circulation rates in the population. Orieux et al. [11] analysed the GAS-associated ICU admissions to several hospitals in France, where they found a higher proportion of influenza co-infections. Therefore, a possible explanation might be that the recurrence of viral infections, for which protection was also previously provided by masks and contact restrictions, also favours a rise in GAS infections [11, 12]. However, this would most likely not explain the increase in non-respiratory infections, such as wound infections caused by GAS. In addition, the exposure to GAS was reduced during the pandemic, in particular in children, which might be associated with decreased immunity to GAS in the overall population and consequently higher susceptibility after relaxation of isolation measures [13]. Moreover, there is evidence for an emergence of specific emm-types [14]. Whole-genome sequencing of GAS isolates from a hospital in northern Germany also revealed the presence of the hypervirulent M1_UK_ lineage in invasive GAS infections [15]. Interestingly, the increase in GAS detection was observed across all sampled materials and apparent infection severity (invasive vs. non-invasive, ICU vs. non-ICU), indicating that the range of clinical manifestations has not changed and that a sampling bias can unlikely explain the increase of cases. Our study emphasizes the need for awareness of the ongoing increased incidence of (i)GAS in adults, as a return to pre-pandemic levels is not yet certain. Follow-up studies will be required to understand the development in coming years and to evaluate long-term effects of the pandemic.

### Limitations of the study

We performed a monocentric retrospective study with a focus on microbiologic GAS detections. Moreover, clinical details such as infection focus, comorbidities, or severity scores were not available in a structured digital format. To mitigate this limitation, we carefully assessed microbiological materials, distinguishing invasive from non-invasive samples, Additionally, transferred patients with external GAS confirmation may have been missed, potentially underestimating incidence. The analysis of positivity rates supports a genuine rise in GAS detections; however, potential shifts in clinical sampling practices over time cannot be fully ruled out. In addition, the isolates were not available for systematic serotyping.

## Data Availability

Due to patient privacy protections, the datasets analyzed during the study are notaccessible to the public.

## References

[1] Gentile A, Juárez M, del Ensinck V, Lopez G, Melonari O, Fernández P. Comparative analysis of influenza epidemiology before and after the COVID-19 pandemic in Argentina (2018–2019 vs. 2022–2023). Influenza Other Respir Viruses. 2025;19:e70078. 10.1111/irv.70078. 39962921 10.1111/irv.70078 PMC11832905

[2] Maison N, Omony J, Rinderknecht S, Kolberg L, Meyer-Bühn M, von Mutius E, et al. Old foes following news ways?—Pandemic-related changes in the epidemiology of viral respiratory tract infections. Infection. 2023. 10.1007/s15010-023-02085-w. 37644253 10.1007/s15010-023-02085-w PMC10811157

[3] Geneva. World Health Organization. Global tuberculosis report 2024. 2024. Licence: CC BY-NC-SA 3.0 IGO.

[4] Sauteur PMM, Beeton ML, Pereyre S, Bébéar C, Gardette M, Hénin N, et al. Mycoplasma pneumoniae: delayed re-emergence after COVID-19 pandemic restrictions. Lancet Microbe. 2024;5:e100–1. 10.1016/S2666-5247(23)00344-0. 38008103 10.1016/S2666-5247(23)00344-0

[5] Brouwer S, Rivera-Hernandez T, Curren BF, Harbison-Price N, De Oliveira DMP, Jespersen MG, et al. Pathogenesis, epidemiology and control of group A Streptococcus infection. Nat Rev Microbiol. 2023;21:431–47. 10.1038/s41579-023-00865-7. 36894668 10.1038/s41579-023-00865-7 PMC9998027

[6] Mohapatra RK, Kutikuppala LVS, Mishra S, Tuglo LS, Dhama K. Rising global incidence of invasive group A Streptococcus infection and Scarlet fever in the COVID-19 era – our knowledge thus Far. Int J Surg. 2023;109:639–40. 10.1097/JS9.0000000000000232. 37093100 10.1097/JS9.0000000000000232 PMC10389320

[7] de Gier B, Marchal N, de Beer-Schuurman I, Te Wierik M, Hooiveld M, Study Group ISIS-AR, et al. Increase in invasive group A Streptococcal (Streptococcus pyogenes) infections (iGAS) in young children in the Netherlands, 2022. Euro Surveill. 2023;28:2200941. 10.2807/1560-7917.ES.2023.28.1.2200941. 36695447 10.2807/1560-7917.ES.2023.28.1.2200941 PMC9817208

[8] Holdstock V, Heppenstall E, Corrigan D, Eccleston A, Jones L, Kalima P, et al. National 10-year cohort study of Life-threatening invasive group A Streptococcal infection in Children, 2013–2023. Pediatr Infect Dis J. n.d. 10.1097/INF.0000000000004855. 10.1097/INF.0000000000004855. PMC12422610 40359236

[9] Leśnik P, Janc J, Biała M, Bartoszewicz M, Łysenko L, Słabisz N. Old Bug-New challenges after COVID-19 pandemic: severe invasive Streptococcus pyogenes infections in Adults-A Single-Center experience in Poland. Pathogens. 2025;14:199. 10.3390/pathogens14020199. 40005574 10.3390/pathogens14020199 PMC11857883

[10] Mutevelli J, Singer R, Dörre A, Feig M, Noll I, Eckmanns T et al. Epidemiologie der Gruppe-A-Streptokokken in Deutschland, 2023–2024 2024. 10.25646/12000

[11] Orieux A, Prevel R, Dumery M, Lascarrou J-B, Zucman N, Reizine F, et al. Invasive group A Streptococcal infections requiring admission to ICU: a nationwide, multicenter, retrospective study (ISTRE study). Crit Care. 2024;28:4. 10.1186/s13054-023-04774-2. 38167516 10.1186/s13054-023-04774-2 PMC10759709

[12] Mania A, Mazur-Melewska K, Witczak C, Cwalińska A, Małecki P, Meissner A, et al. Invasive group A Streptococcal infections as a consequence of coexisting or previous viral infection in the post-COVID-19 pandemic period. J Infect Public Health. 2025;18:102622. 10.1016/j.jiph.2024.102622. 39708759 10.1016/j.jiph.2024.102622

[13] Abo Y-N, Oliver J, McMinn A, Osowicki J, Baker C, Clark JE, et al. Increase in invasive group A Streptococcal disease among Australian children coinciding with Northern hemisphere surges. Lancet Reg Health West Pac. 2023;41:100873. 10.1016/j.lanwpc.2023.100873. 38223399 10.1016/j.lanwpc.2023.100873 PMC10786649

[14] Davies MA, de Gier B, Guy RL, Coelho J, van Dam AP, van Houdt R, et al. Streptococcus pyogenes Emm type 3.93 Emergence, the Netherlands and England. Emerg Infect Dis. 2025;31:229–36. 10.3201/eid3102.240880. 39983683 10.3201/eid3102.240880 PMC11845126

[15] Wolters M, Berinson B, Degel-Brossmann N, Hoffmann A, Bluszis R, Aepfelbacher M, et al. Population of invasive group A Streptococci isolates from a German tertiary care center is dominated by the hypertoxigenic virulent M1UK genotype. Infection. 2024;52:667–71. 10.1007/s15010-023-02137-1. 38064158 10.1007/s15010-023-02137-1 PMC10954911

